# O1.8. STRESS-INDUCED AMYGDALA HYPERACTIVITY LEADS TO INTERNEURON LOSS AND SCHIZOPHRENIA-LIKE PATHOLOGY IN A DEVELOPMENTAL DISRUPTION MODEL OF SCHIZOPHRENIA

**DOI:** 10.1093/schbul/sby015.190

**Published:** 2018-04-01

**Authors:** Anthony Grace, Felipe Gomes, Xiyu Zhu

**Affiliations:** University of Pittsburgh

## Abstract

**Background:**

Studies of the MAM rat model of schizophrenia show that rats exhibit higher anxiety levels, greater response to stress, and amygdala hyperactivity prepubertally before the emergence of the hyperdopaminergic state later in life. Furthermore, administration of diazepam prepubertally prevents this transition. These data suggest that MAM may predispose to psychosis via increased response to environmental stressors. If this is accurate, then one would predict that sufficiently strong stressors administered to normal rats during the critical period prepubertally would lead to psychosis-like state in the adult.

**Methods:**

Rats are exposed to either 3 sessions of 1-hour restraint, 25 footshocks daily for 10 days, or both stressors delivered either at PD 31–40 (prepuberty) or PD 65–74 (adult) in intact rats and rats with prelimbic PFC (plPFC) lesions at PD25. Rats were tested for amphetamine locomotion, novel object recognition (NOR), and VTA DA neuron firing. Valproic acid (VPA) was administered to adults 5 days before and during the combined stressors to reopen the critical period. DREADD activation of the amygdala was also evaluated.

**Results:**

While individual stressors prepubertally augmented anxiety and disrupted NOR in the adult, only the combined stressors resulted in amphetamine hyperlocomotion and increased DA neuron population activity similar to the MAM rats. plPFC lesions enabled the footshock alone to lead to anxiety, NOR deficits, and the hyperdopaminergic state in the adult. Given that stress is known to impact parvalbumin (PV) interneurons in the hippocampus when administered during the critical period in prepubertal rats, we tested the impact of opening the critical period in the adult rats with VPA. Normal rats given combined stressors at PD 65–74 showed attenuated DA neuron activity similar to that observed in depression models. However, administration of VPA caused the combined stressors to lead to the schizophrenia phenotype. This was accompanied by hippocampal hyperactivity driven by an overactive amygdala, since DREADD activation of the amygdala produced similar effects.

**Discussion:**

These data suggest that factors that increase the response to environmental stressors during the prepubertal critical period lead to activation of the stress-activated amygdala-hippocampal pathway and PV interneuron loss, which leads to the hyperdopaminergic state in the adult thought to underlie psychosis. Furthermore, re-opening the critical period in the adult makes the adult sensitive to stress-induced psychosis. This suggests that controlling the impact of stress early in life in susceptible individuals may be an effective means to circumvent the transition to psychosis later in life.

